# Revisiting the Relationship between Altruism and Organ Donation: Insights from Israel

**DOI:** 10.3390/ijerph19127404

**Published:** 2022-06-16

**Authors:** Keren Dopelt, Lea Siton, Talya Harrison, Nadav Davidovitch

**Affiliations:** 1 Department of Public Health, Ashkelon Academic College, Ben Tzvi St. 12, Ashkelon 78211, Israel; dopelt@bgu.ac.il (K.D.); leasiton@edu.aac.ac.il (L.S.); talya1@edu.aac.ac.il (T.H.); 2 Department of Health Policy and Management, School of Public Health, Faculty of Health Science, Ben Gurion University of the Negev, P.O. Box 653, Beer Sheva 84105, Israel

**Keywords:** organ donation, altruism, behavioral intentions, transplantation, religiosity

## Abstract

The number of people on the waiting list for an organ transplant increases year after year. However, the number of donated organs available for transplantation does not rise in line with this increased demand. This study examines the associations between altruism, attitudes towards organ donation, and behavioral intentions regarding organ donation within the Jewish population in Israel. In a cross-sectional study, 452 participants completed an online questionnaire. Data collection occurred between November and December 2020. Convenience sampling was used, and participation was voluntary. Data were analyzed using Pearson correlations and independent samples *t*-tests. Within the study population, we found high levels of altruistic behaviors and positive attitudes toward organ donation. However, the level of behavioral intentions toward organ donation was low. No associations were found between altruism levels and attitudes toward organ donation, or between altruism levels and the degree of behavioral intentions toward organ donation. However, a positive relationship was found between attitudes toward organ donation and willingness to sign an organ donor card. In addition, positive associations were found between religiosity and altruism, while negative associations were found between religiosity and attitudes towards organ donation, and between religiosity and willingness to sign an organ donor card. Positive attitudes toward organ donation may result in increased organ donation in the future. Thus, raising awareness and positive attitudes toward organ donation among the wider public and, in particular, the ultra-Orthodox population in Israel in particular is necessary. Consequently, it is essential that information about the organ donation process is accessible and culturally adaptive to different sectors.

## 1. Introduction

Organ transplantation is one of the clearest examples of how technological advancement has enabled the realization of ancient medical ideas. The possibility of transplanting an organ from a healthy person into the body of a sick person has captured the medical imagination for many centuries. However, it was only in the second half of the twentieth century that this vision became a reality, with the first kidney transplants in the 1950s followed by pancreas, liver, and heart transplants in the 1960s. Since then, technological development has enabled more organs and tissues to be transplanted, and organ transplants have become a viable solution for a growing number of medical conditions [1].

Since the outset of the development of transplant medicine, ethical rules have been established for the supply of human organs for transplantation. Above all, such organs must be obtained through altruistic donation, i.e., given freely without any material consideration. The source of the altruistic rule in organ donation can be found in the revolution that inspired Richard Titmuss’s book *The Gift Relationship* [2]. Titmuss compared blood collection systems around the world and concluded that voluntary blood donation is the most effective, safe, and ethical method for collecting blood. According to Titmuss, voluntary or altruistic donations express a pure desire to help and, therefore, such donors are free from fears of fraud or falsification of medical data. Further, they contribute to social solidarity and are a buffer against trends in commercializing human relationships.

This view is the dominant paradigm when it comes to collecting human cells, tissues, and organs for therapeutic purposes. Although Titmuss’s book is outdated in many ways, the paradigm he proposed still prevails as the ethical boundary when it comes to organ transplantation. For Titmuss, an individual’s altruistic behavior is the driving force behind the mechanism of organ donation. Altruistic individuals create a norm of volunteering and donating that, in turn, increases social solidarity. The state should create “opportunities for altruism” for individuals, such as, for example, the opportunity to donate blood or organs for transplantation. The aggregated altruistic behavior of individuals crystalizes into a social norm, contributes to social solidarity, and, in a virtuous circle, leads to an increase in altruistic behaviors. This position forms the basis for policies for collecting organs for transplantation and underpins advocacy systems for encouraging organ donation around the world. The assumption is that altruism constitutes an extant force in society and can be mobilized as a solid base for organ collection policies.

Generally, deceased organ procurement schemes follow “opt-in” or “opt-out” models. In opt-in or informed consent models, organs are procured from deceased people who carried organ donor cards or any other form of personal statement of their will to donate their organs after death. These models are the current policy in Israel and in the US, as well as in other countries. In an opt-out system, organs are procured under the presumption that an individual consents to become an organ donor after death, unless that person has explicitly opted out of the system. Such a model is more widespread in Europe, with the UK, France, and Switzerland joining Spain, Germany, Austria, and other nations in their opt-out donation policies. There is an indication in most research that opt-out models yield more donations [3]. In Israel, the current policy is opt-in [4].

The impact of religion on organ donation is positive [5,6]. However, there is controversy within orthodox Judaism regarding deceased organ donations after brain death [7]. Islam also has a rather supportive stance towards organ donations [8], but research indicates a that religious stance toward organ donation is inseparable from cultural and social standing [9].

This paper examines the assumption that altruism is associated with organ donation in Israel and helps to shed light on the complexity of this concept. The paper also highlights the need for a broad cultural and social context if the complex interplay between social structures and organ donation is to be understood.

### 1.1. Quantifying Altruism

Altruism is defined as behavior aimed at helping others [10]; it may promote prosocial behavior that is sensitive to the other people’s actual needs [11]. The behavior is carried out even when the helper does not expect any benefit or return, or when the behavior may endanger the helper to some extent [12]. On an extreme level, altruism can manifest itself in conscious self-sacrifice for the sake of others. Altruism is also defined as the social motive for doing good [13]. 

Various scales for measuring altruism are described in the literature. For example, the “dictator game” examined altruistic charitable donation through a survey or experiment [14,15,16]. Sliwak developed the Altruism-Nonaltruism (A-N) Questionnaire, which contains ten stories, each with six answers that reflect the various degrees of intensity of a person’s altruistic attitude [17]. Another method measures the orientation to altruistic values through self-rating on an altruistic value questionnaire, focusing on concepts such as fairness, world peace, and social justice [18]. 

The most common scale for measuring altruistic behavior is Rushton’s Self-Report Altruism (SRA) scale. Rushton et al. [19] developed a set of 20 statements to measure the level of helping or altruistic personality traits. For example, “I have helped push a stranger’s car out of the snow”; “I have given directions to a stranger”; “I have given money to a charity”; “I have given money to a stranger who needed it”; “I have donated goods or clothes to a charity”; “I have donated blood”; “I have helped carry a stranger’s belongings (books, parcels, etc.)”. Respondents were asked to mark how often they had participated in each behavior, ranging from never (1) to very often (5). Rushton’s scale [19] been translated and validated in many languages and cultures, including Mandarin [20], Hindi [21], Spanish [22], Indonesian [23], Turkish [24], Dutch [25], and Hebrew [26].

### 1.2. The Relationship between Altruism and the Willingness to Donate Organs

Many studies use altruism as a predictor of organ donation. Khalaila [26] used Rushton’s SRA scale [19] and found that Israeli students’ willingness to donate organs was positively related to their altruism level, their positive attitudes toward organ donation, and their organ donor registration. However, the students’ knowledge level had no impact on their willingness to donate. Further, while students who identified as Christian were more willing to donate organs than students of other religions in Israel, religiosity was negatively associated with behavioral intentions regarding organ donation. Milaniak et al. [17] examined the role of empathy and altruism in organ donation decision-making among 111 nursing and paramedic students, using the Sliwak Altruism scale. They found that altruism levels were associated with post-mortem organ donation and the willingness to sign a donor card.

In a meta-analysis that included 27 studies (most of them based on semi-structured interviews and focus groups), altruistic motivation to help others emerged as the most-identified motivator for becoming an organ donor. Altruistic motivation to help another person, while knowing that the organs help another person improve their quality of life and longevity, was a driving force behind the consent to donate organs. Other factors included a sense of solidarity with society and a belief that organ donation is used for beneficial purposes with patients [27].

Given the deficiency in the number of organs and, therefore, long waiting times for transplantation (eight years on average in Israel and five years the US), policymakers face the challenge of raising organ donations [28]. Because organ supply depends on public motivation to donate, the roles of altruism and attitudes regarding organ donation are essential when considering the unavailability of organs for transplantation. However, little is known about the impact of these factors on the general population’s willingness to donate in Israel. Previous research performed in Israel was conducted among college students [26], whereas the current research was conducted among the general population. Another study in Israel examined the knowledge and preferences of Israel’s Jewish population regarding the organ allocation policy [28] rather than willingness to donate. An examination of the association between altruism and attitudes would assist in identifying groups in the population that do not tend to sign an organ donation card and in developing culture-adapted interventions and educational programs to increase awareness of organ donation.

Drawing upon the literature on the association between altruism and organ donation, this study examined this relationship in Israeli society, and specifically considered whether altruism levels are linked to organ donation in Israel. We hypothesized that positive associations would be found between altruism, attitudes toward organ donation, and willingness to sign an organ donation card.

## 2. Materials and Methods

Ethical approval for the current study was obtained from the Ethics Comity, Ashkelon Academic College (#26-2020).

### 2.1. Participants and Procedure

The cross-sectional study involved 452 participants from the adult population in Israel who were sampled by way of a convenience sample. The questionnaire was programmed using Qualtrics survey software, and on 14 November 2020 a link to fill out the questionnaire was distributed on social networks (WhatsApp and Facebook groups). A week later, a reminder was sent to the groups, and on 1 December 2020, the survey was closed. According to the survey data, the response time for the questionnaire was, on average, around 4.5 min. The survey contained 564 entries; 463 participants filled out the questionnaire and 11 participants were removed because they failed to fill out the answers for the dependent variable. Therefore, the response rate to the survey was 80% of the total entries. At the beginning of the questionnaire, the purpose of the study was explained. Filling out the questionnaire constituted informed consent to participate in the study and no questions were defined as mandatory. The survey was administered without prior information about donation.

### 2.2. Research Tool 

An online, closed, anonymous, self-report questionnaire was used. For validation purposes, the questionnaire was provided to ten employees at the Ashkelon Academic College between the ages of 25–50 from various departments, different sexes, and different ethnic origins. Four questions were corrected based on written comments made by participants. The questionnaire included 31 closed questions.

1. Demographics: Sex, age, marital status, parental status, level of religiosity, country of birth.
2. Altruistic behavior: The questionnaire included ten questions from Watad [29] that were adapted from Rushton et al. [19]. The questionnaire was translated into Hebrew, adapted to Israeli culture, and validated. Of the 20 items that appeared in Rushton’s questionnaire, 10 items that did not correspond to Israeli culture (for example, “pushing a car stuck in the snow”, as there almost no snow in Israel) were removed, following a content validation process with eight PhD students from the School of Social Work at Tel Aviv University. Furthermore, the full and the shorter versions of the questionnaire were distributed to 40 students from Tel Aviv University, one week apart. Pearson correlation between the versions was r_p_ = 0.79, *p* < 0.001). The short questionnaire was further examined by experts from the Chief Scientific Bureau of the Ministry of Education; based on their comments, the wording of the questionnaire was finalized. The reliability of the translated questionnaire was α = 0.83. Participants were asked to indicate their level of agreement with each statement on a Likert scale ranging from 1 (never) to 5 (often). The statements described everyday acts of altruism, such as “I donated money to charity.” The variables were constructed using a mean calculation for each participant, with a higher score indicating a higher level of altruistic behavior. The internal consistency of the study was α = 0.75. Appendix A provides the distribution of the answers.
3. Attitudes towards organ donation: The questionnaire included 13 questions taken from Utitz [30]. The attitudes questionnaire was based on the literature and validated by seven experts in bioethics and organ donation, using a content validity method. There was agreement between them regarding most of the statements. Three statements that generated disagreement were removed. The reliability of the questionnaire was Cronbach’s α = 0.80. Participants were asked to indicate their level of agreement with each statement in the questionnaire on a Likert scale ranging from 1 (strongly disagree) to 5 (very much agree). The statements related to general attitudes towards organ donation, for example: “Organ donation is a human *mitzvah* (commandment).” The variables were constructed using a mean calculation for each participant after reversing the scales in questions 1, 4, 5, 7, 8, and 10. A higher score indicated more positive attitudes toward organ donation. The internal consistency of the study was α = 0.94. Appendix B provides the distribution of the answers.
4. Behavioral intentions regarding organ donation: Two questions were taken from Utitz [30]. The questions were based on the literature and validated by seven experts in bioethics and organ donation, using a content validity method. There was agreement between them regarding the questions. The reliability was Cronbach’s α = 0.86. Participants were asked to indicate their level of agreement with each statement in the questionnaire on a Likert scale ranging from 1 (not at all) to 5 (very much agree), with an option to mark “I have already signed a donation card.” The variables were constructed using a mean calculation for each participant, with a higher score indicating a higher behavioral intention regarding organ donation. The internal consistency of the study was α = 0.89.

### 2.3. Data Analysis

The data were imported from the survey software and analyzed in SPSS v. 26 (IBM, Armonk, NY, USA). A probing analysis found a normal distribution of the variable, and, therefore, parametric tests were used. The relationships between the variables were examined using Pearson correlations. The relationships between the level of religiosity and the study variables were analyzed using Spearman correlations. Differences between categories (e.g., having signed a donation card, having children) were tested using independent samples t-tests. A linear regression model was used to test the prediction of willingness to donate. The independent variables were altruism and religiosity. All reported *p* values were based on two-sided tests and were considered significant when the values were below 0.05.

## 3. Results

### 3.1. Sample Characteristics

In total, 452 individuals participated in the study, of whom 72.1% were women, 60% were married, and 48.2% had children. Most of the participants were Israeli born (90%). About a quarter of the participants defined themselves as secular, 23.7% as traditional, 42.9% as religious, and 8.6% as ultra-Orthodox. Sample characteristics are shown in Table 1.

### 3.2. Willingness to Donate Organs/to Sign an Organ Donor Card

In response to the statement, “I would like to donate organs after my death”, 30% of participants selected a low level of agreement (responses 1 and 2), 21% selected a medium level of agreement (response 3), and 49% chose a very high level of agreement (responses 4 and 5). A quarter of the sample (*n =* 111, 26%) had already signed an organ donor card. The remaining respondents (*n =* 341) responded to the statement, “I am considering signing an organ donor card,” as follows: 50% chose a low level of agreement (responses 1 and 2), 21% chose a medium level (response 3), and 29% chose a high level (responses 4 and 5). The mean for the variable “willingness to donate/sign an organ donor card” was 3.21 (SD = 1.52).

### 3.3. Relationships between the Study Variables

No relationship was found between altruism and attitudes toward organ donation, or between altruism and the level of willingness to donate/sign an organ donor card, as the results were not significant (*p* > 0.05). Thus, the hypotheses in that regard were not confirmed. A positive, significant association was found between attitudes toward organ donation and the degree of willingness to donate/sign an organ donor card (r_p_ = 0.86, *p* < 0.001). That is, the more positive the attitudes toward organ donation, the higher the willingness to donate/sign an organ donor card. Thus, the hypothesis in that regard was confirmed.

### 3.4. Association between Age and the Study Variables

Positive, significant, weak-to-moderate associations were found between age and altruism (r_p_ = 0.16, *p* ≤ 0.01), between age and the attitude toward organ donation (r_p_ = 0.20, *p* < 0.001), and between age and the level of willingness to donate/sign an organ donor card (r_p_ = 0.19, *p* < 0.001). The older the respondents, the more altruistic they were, the more positive their attitudes were toward organ donation, and the higher their willingness to donate/sign an organ donor card.

### 3.5. Associations between Levels of Religiosity and the Study Variables

A positive, significant, weak-to-moderate association was found between level of religiosity and altruism (r_s_ = 0.15, *p =* 0.001). Further, negative and significant moderate-to-high associations were found between level of religiosity and (a) attitude toward organ donation (r_s_ = 0.36, *p* < 0.001) and (b) level of willingness to donate/sign an organ donor card (r_s_ = 0.32, *p* < 0.001). That is, as the religiosity level increased, the respondents were more altruistic, their attitudes toward organ donation were more negative, and their willingness to donate/sign an organ donor card was lower.

Table 2 shows the association between altruism and willingness to donate/sign an organ donor card in each category of religiosity.

A positive, significant, moderate association was found among the secular group participants between altruism and willingness to donate/sign an organ donor card (r_p_ = 0.20, *p* < 0.05). No associations were found in the other categories of religiosity.

In a linear regression that tested the prediction of willingness to donate, a significant predictive ability of the level of religiosity was found (β = −0.33, B = −0.53, *p* < 0.001), whereas the level of altruism did not make a significant contribution to the model (β = 0.08, B = 0.18, *p* > 0.05). The model was significant (F_(451)_ = 27.08, *p* < 0.001, Adj R^2^ = 0.10).

### 3.6. Differences between Those Who Had Signed and Those Who Had Not Signed an Organ Donor Card

Statistically significant differences were found between respondents who had signed an organ donor card and respondents who had not signed an organ donor card (t_(450)_ = 17.57, *p* < 0.001). Those who had signed an organ donor card had more positive attitudes toward organ donation than those who had not signed an organ donor card (mean of 4.51 compared to 3.29). In terms of levels of altruism, no differences were found between respondents who had signed an organ donor card and respondents who had not signed an organ donor card. 

### 3.7. Differences between Respondents Who Had Children and Respondents Who Did Not Have Children

In terms of altruism levels, statistically significant differences were found between respondents with children and respondents without children (t_(450)_ = 4.76, *p =* 0.001). Respondents who had children expressed higher levels of altruism compared with those who did not have children (mean of 3.43 vs. 3.12). Further, there were significant differences between respondents with children and respondents without children in terms of attitudes toward organ donation (t_(450)_ = 2.06, *p =* 0.05). Respondents with children had more positive attitudes toward organ donation than those without children (mean of 3.70 vs. 3.49). In terms of willingness to donate organs/sign an organ donor card, no differences were found between respondents with children and respondents without children. 

### 3.8. Differences between Relationship Status

In terms of altruism levels, significant differences were found between different types of relationship status (t_(446)_ = 4.71, *p* < 0.001). Respondents who were married or living with a spouse expressed higher levels of altruism, compared with others (single, divorced/separated, widower) (mean of 3.40 vs. 3.09). In terms of attitudes toward organ donation, no significant differences were found between married respondents, respondents living with a spouse, and other types of relationship status (single, divorced/separated, widower) (t_(446)_ = 0.18, *p =* 0.856, mean of 3.60 vs. 3.58, respectively). In terms of willingness to donate organs/sign an organ donor card, no significant differences were found between married respondents, respondents living with a spouse, and other types of relationship status (single, divorced/separated, widower) (t_(446)_ = 0.75, *p =* 0.453, mean of 3.17 vs. 3.28, respectively).

### 3.9. Differences between the Sexes

No significant differences were found between males and females in terms of altruism levels (t_(450)_ = 1.25, *p =* 0.211, mean of 3.34 vs. 3.25, respectively), attitudes toward organ donation (t_(450)_ = 0.46, *p =* 0.645, mean of 3.63 vs. 3.58, respectively), and willingness to donate organs/sign an organ donor card (t_(450)_ = 0.27, *p =* 0.784, mean of 3.24 vs. 3.20, respectively).

## 4. Discussion

This study sought to investigate the levels of willingness in the general population in Israel to sign an organ donor card, the relationship between attitudes toward organ donation and altruism, and the relationship between attitudes toward organ donation and willingness to sign an organ donor card. The level of altruism among participants in the study was high (mean 3.28 on a 5-point scale); the attitudes toward organ donation were positive (mean 3.60 on a 5-point scale); and willingness to donate organs was high (mean 3.21 on a 5-point scale). A quarter of respondents had already signed an organ donor card. However, the distribution of responses to the statements revealed a complex picture: while the majority of respondents (68%) perceived organ donation as important, as having exemplary moral value, and as a human *mitzvah* (“commandment”), only 55% were in favor of signing an organ donor card. Among respondents who believed that organ donation is a moral obligation, the study found an even lower percentage, 41%, with only 26% showing a real commitment to organ donation through signing an organ donor card. This large gap between explicit attitudes and actual commitment to donate organs may be due to religious beliefs. These findings reinforce the findings of previous studies, where similar gaps were found between intent and real actions [31]. Even with voluntary blood donation, an action that is far simpler than organ donation, there is an enormous gap between willingness to donate (90%) and actually taking action to donate (only 20%) [32]. 

There are numerous fears that prevent people from signing an organ donor card, including fear of bodily “mutilation” after death and anxiety issues concerning death. Indeed, only 58% of respondents agreed with the statement on the questionnaire, “The thought that I will be cut open after my death does not deter me”. Death and post-mortem organ donation are closely linked, as registering as an organ donor card holder requires an individual to recognize that he or she is “mortal.” Thus, our findings are consistent with studies that found that anxieties around death can intensify and be triggered when people are considering decisions regarding organ donation [33,34].

In the entire sample, no association was found between altruism and attitudes regarding organ donation, or between altruism and willingness to donate organs in practice. These findings are not consistent with previous studies [35,36,37,38,39]. However, the findings can be explained by reference to the literature concerning the numerous considerations for organ donation (e.g., mourning for the deceased and anxieties) that overcome altruistic values [40,41]. There seems to be a considerable distinction between altruistic behavior and signing an organ donor card, and even more of a distinction when it comes to donating an organ (e.g., a kidney) in an altruistic living donation. Whereas an altruistic person is expected to consider others, and to consider the demand for organ donation, organ donation is not a common act of assistance such as donating money or other aid; rather, it is a very complex emotional process. Indeed, organ donation is almost entirely unlike any other behavior undertaken for the good of others. The regression model showed that the level of religiosity was the most strongly predictive of willingness to sign a donation card, while altruism itself was not a significant predictor. People with a higher level of religiosity were less willing to sign a donation card, which meant that religiosity level was a major barrier to future organ donation, regardless of the level of altruism.

Examining the relationship between the variables according to religiosity levels, we found that there is no association between altruism and willingness to donate organs in the groups of traditional, religious, and ultra-Orthodox participants; however, we found a positive association between the variables among the secular participants. This finding underscores the importance of perceptions of Jewish law at their extreme level. Secular Jews generally do not delve into ways of determining death under Jewish law and, therefore, have no ideological-religious problem with organ donation.

A positive, strong association was found between attitudes toward organ donation and willingness to donate organs in practice, a finding that was similar to findings in previous studies [42,43,44,45]. Positive attitudes regarding the topic of organ donation have a positive effect on peoples’ willingness to be future organ donors. This finding suggests that shaping public opinion so that organ donation is perceived as positive may lead to an increase in willingness to donate organs in the future and could encourage people to sign organ donor cards now. In their Theory of Reasoned Action (TRA), Fishbein and Ajzen [46] argued that the intention to carry out a behavior is the best predictor of its occurrence, and that this is dependent on attitudes and norms held by the individual. An individual’s positive attitudes, together with social norms that call for saving human lives through organ donations, gives rise to a social process that reinforces these values. This process reinforces motivations and intentions to sign organ donor cards for the purpose of saving human lives.

Another point that emerged from this study was that the older the respondents, the more altruistic they were, the more positive their attitudes were toward organ donation, and the greater their willingness was to donate/sign organ donor cards. It may be that as a person grows older, and his or her convictions become stronger, he or she understands how precious life is and becomes more exposed to cases of illness. Among younger age groups, the issue of organ transplants may seem like a topic that is not relevant to them, and they may not have given sufficient thought to its importance, as Krupic et al. [47] and Febrero et al. [48] found. It should also be noted, however, that the attitudes of young people toward organ donation are important, because from a medical perspective they are ideal donors, as their organs are less likely to be damaged by poor health.

It also emerged from this study that respondents with children were characterized by a higher level of altruism and more positive attitudes toward the topic of organ transplantation. Again, this group may comprise older individuals whose views on life in general, and specifically on the issue of organ transplants, differ from those of younger people who are not at a stage of life where they have children.

When it comes to the level of religiosity and its association with the study variables, a contradiction emerged: as the level of religiosity rose, so did the level of altruism, However, at the same time, willingness to donate organs decreased. Previous findings regarding the relationship between the level of religiosity and willingness to donate organs were not unequivocal. Past findings in other countries indicated that religiosity negatively impacts donation decisions, e.g., such findings were made in Spain [49], Sweden [47], the USA [50], and in Israel [26]. However, a study that investigated organ donation for participants in China and the United States showed that altruism and religiosity were not significant predictors of a willingness to donate organs [51]. Yoon [52] suggested that a high level of religiosity indicates a high willingness to donate organs.

The reasons for this are varied and lie mainly in the *halakhic* (Jewish law) aspects of organ donation after death. In ultra-Orthodox Judaism, there is a debate regarding the validity of the definition of brain death. The conservative point of view holds that the pulse is the ultimate measurement, even though the brain has stopped functioning. A second approach argues that brain death can be recognized as a condition for determining death, because the person cannot breathe independently. A third attitude accepts brain death as a determinant of death. Many groups within the ultra-Orthodox Israeli Jewish population do not sign organ donor cards or donate organs because of their rabbis’ opposition to the definition of brain death. However, in 1982 the Rabbinate of Israel adopted the third approach, that brain death is a determinant of death. Therefore, according to Jewish law, organ donation is permitted [53].

In this context, it is worth noting that despite their reluctance to donate organs after death, the ultra-Orthodox are the largest group in terms of living organ donations. This group leads in the donations of organs from living people to total strangers. This behavior, which is certainly altruistic, is carried out by a group that in this study was found to be distant from engaging in organ donations.

The willingness to donate organs can be equated with attitudes toward euthanasia, because both issues are moral and related to end-of-life decisions, and both issues are controversial in the context of Judaism. Dopelt et al. [54] examined attitudes toward euthanasia among physicians working at a tertiary-care hospital in Israel. They found a negative relationship between the level of religiosity and attitudes toward euthanasia. Moreover, in a linear regression analysis, the level of religiosity was the best predictor of attitudes toward euthanasia. This example also illustrates the dominance of Jewish law in Israel.

The trend among the ultra-Orthodox communities in Israel in donating organs to strangers indicates the need for future studies to differentiate between motivations for post-mortem donations and living organ donations. While signing an organ donor card indicates a contribution to the community, living organ donation usually occurs within families to help a sick relative. These are two types of altruistic acts that require different definitions and different operationalizations.

### Study Limitations

This paper has several important limitations. The study sample was limited and did not represent the entirety of the Israeli population (e.g., the sample did not include any representation from Arab Muslim, Christian, or Druze populations). We focused on the Jewish population based on the assumption that religious beliefs and cultural differences might affect attitudes and viewpoints. In addition, the sample comprised mostly women (72%), and the ultra-Orthodox Jewish religious populations were over-represented relative to the general population. Further, there may have been biases in the study, such as a selection bias in response to the questionnaire, with the result that those who chose to respond may have had a special interest in the issue of organ donation. An additional bias was social desirability—some participants may have provided answers to questions on the attitudes and barriers questionnaire that they thought were expected of them and selected more positive or altruistic behaviors. An additional problem was the tools that were used for measuring altruism. The questionnaire examined altruistic behaviors that almost all people carry out by virtue of being social beings, but altruism should be examined as a personality trait and not as a behavior.

## 5. Conclusions

In Israel, the current supply of human organs for transplantation is far less than the demand. Broadly, the reason for this is twofold: the Jewish-*halakhic* (Jewish law)-religious issue and a lack of public awareness and positive incentives for organ donation. The positive association between attitudes and willingness to donate organs shows that positive attitudes toward organ donation may ultimately be translated into donations in the future. For this reason, increasing positive attitudes within the population is an important aspect of organ donation, because the formation of a positive approach is a critical step in making decisions regarding this complex topic, particularly if the aim is to reduce the number of people who oppose organ donation in Israel.

It is important to note the difference between willingness to donate organs to a stranger after death and willingness to donate organs to a stranger while alive. This difference is especially significant in relation to the study’s findings among the ultra-Orthodox populations in Israel. The data indicated a high level of altruism and a low level of willingness to donate organs within this population; however, these data should be interpreted with caution, because the low levels of willingness to donate organs relate to post-mortem organ donation, whereas there is a high level of willingness within this population for living organ donation to strangers, which is the most altruistic act of organ donation.

## Figures and Tables

**Table 1:** Sample characteristics (*n =* 452).

Characteristics	*n*	%
Male	126	27.9
Female	326	72.1
Marital status:		
Married, living with a spouse	271	60.0
Single	151	33.4
Divorced/separated	23	5.1
Widower	3	0.7
Level of religiosity:		
Secular (don’t live by the Jewish law at all)	112	24.8
Traditional (Conservative. Adopts some Jewish law, usually going to synagogue on Saturdays and holidays)	107	23.7
Religious (Maintains a religious lifestyle according to Jewish law, goes to synagogue every day)	194	42.9
Ultra-Orthodox (Very religious. Conservatively adhering to Jewish law, studying Torah every day all day)	39	8.6
Having children	218	48.2
Country of Birth:		
Israel	407	90
Former USSR countries	31	6.9
Western Europe	10	2.2
Ethiopia	2	0.4
USA	2	0.4
Age (Range: 18–76, Mean: 32 ± 11.60)		
18–25	118	34.6
26–35	114	33.4
36–45	58	17.0
45+	51	15.0

**Table 2:** The association between altruism and willingness to donate/sign an organ donor card in each category of religiosity.

	r_p_	*p*
Secular	0.20	0.032
Traditional	0.11	0.119
Religious	0.03	0.768
Ultra-Orthodox	0.04	0.811

## Data Availability

The data that support the findings of this study are available from the first author upon request.

## Appendix A. Distribution of Responses to the Altruistic Behavior Questionnaire

Table A1 shows the distribution of responses to the statements examining altruistic behavior (rating the frequency of behaviors on a five-point scale), after grouping into categories as follows: responses 1 + 2 were grouped into the category “infrequently,” response 3 remained as “more than once,” and responses 4 + 5 were grouped into the category “frequently”.

**Table A1:** Distribution of responses to the altruism questionnaire.

Statement	Infrequently (%)	More than Once (%)	Frequently(%)	Mean ± SD
1. I donated money to a charitable cause	13	14	73	4.08 ± 1.12
2. I gave up my place in line to another person (in the store, cinema, clinic)	10	22	68	3.96 ± 1.02
3. I offered my seat (on the bus etc.) to another person	14	18	68	3.92 ± 1.14
4. I gave money to someone who needed it or who asked me	19	23	58	3.70 ± 1.84
5. I helped someone I don’t know very well carry something heavy	30	23	47	3.37 ± 1.32
6. I helped a person with disabilities/an older person cross the road or carry a bag	33	28	39	3.14 ± 1.27
7. I volunteered to watch a pet or child for a neighbor	51	19	30	2.63 ± 1.43
8. I undertake volunteering activities	52	20	28	2.75 ± 1.39
9. I donated something that was of value to me to another person	47	28	25	2.71 ± 1.22
10. I helped an acquaintance move to a new home	55	23	22	2.45 ± 1.31

To construct the variable “altruism,” the mean value of the responses for each participant was calculated to be 3.28 (SD = 0.70).

## Appendix B. Distribution of Responses in the Attitude Questionnaire Regarding Organ Donation

Table A2 shows the distribution of responses to statements examining attitudes around organ donation (rating agreement on a five-point scale), after grouping into categories as follows: responses 1 + 2 were grouped into the category “slightly agree,” response 3 remained “somewhat agree,” and responses 4 + 5 were grouped into the category “strongly agree.”

**Table A2:** Distribution of responses to the questionnaire regarding attitudes to organ donation.

Statement	Slightly Agree (%)	Somewhat Agree (%)	Strongly Agree (%)	Mean ± SD
1. Organ donation is [is not] contempt for the dead *	16	12	72	4.07 ± 1.39
2. Organ donation is a human *mitzvah*	16	16	68	3.96 ± 1.31
3. Giving life after death by donating organs is an honor	17	17	66	3.86 ± 1.36
4. Organ donation is [is not] desecration of the dead *	18	16	66	3.36 ± 1.39
5. Organ donation is [is not] intervening in the affairs of God *	18	17	65	3.81 ± 1.41
6. I am in favor of organ donation	23	17	60	3.69 ± 1.44
7. Signing an organ donor card will hurt [will not hurt] my family’s feelings *	25	17	58	3.56 ± 1.49
8. The thought of being cut open after my death makes me [does not make me] hesitant *	27	15	57	3.59 ± 1.55
9. I am in favor of signing an organ donor card	27	18	55	3.53 ± 1.49
10. Donating organs makes it difficult [does not make it difficult] for the family after the death of a loved one *	26	25	49	3.36 ± 1.39
11. Signing an organ donor card will make my family think I did a good deed	24	28	48	3.39 ± 1.39
12. Donating organs is a moral duty	33	26	41	3.14 ± 1.47
13. Donating organs will help the family of the deceased cope better with the bereavement	35	34	31	2.93 ± 1.30

* Reverse questions. The data are shown following the reversal of the scales, so the parentheses have been added to reverse the meaning of the statement.

To construct the variable “attitudes toward organ donation,” the mean value of the responses for each study participant was calculated after reversing the scales in the reverse questions. The mean was 3.60 (SD = 1.08).

## References

[1] Barr J., Bradley J.A., Hamilton D., Hakim N., Haberal M., Maluf D. (2021). Organ Transplantation: A Historical Perspective. Transplantation Surgery.

[2] Titmuss R. (1970). The Gift Relationship: From Human Blood to Social Policy.

[3] Madden S., Collett D., Walton P., Empson K., Forsythe J., Ingham A., Morgan K., Murphy P., Neuberger J., Gardiner D. (2020). The effect on consent rates for deceased organ donation in Wales after the introduction of an opt-out system. Anaesthesia.

[4] Boas H., Lavi S., Boas H., Davidovitch N., Filc D., Hashiloni-Dolev Y., Lavi S. (2018). Organ donation, brain death and the limits of liberal bioethics. Bioethics and Biopolitics in Israel: Socio-Legal, Empirical and Political Analysis.

[5] Daar A.S. (1994). The body, the soul and organ donation: Beliefs of the major world religions. Nefrologia.

[6] Oliver M., Woywodt A., Ahmed A., Saif I. (2011). Organ donation, transplantation and religion. Nephrol. Dial. Transplant..

[7] Halevy A., Brody B. (1993). Brain death: Reconciling definitions, criteria, and tests. Ann. Intern. Med..

[8] Rady M.Y., Verheijde J.L. (2014). The moral code in Islam and organ donation in Western countries: Reinterpreting religious scriptures to meet utilitarian medical objectives. Philos. Ethics Hum. Med..

[9] Alkhawari F.S., Stimson G.V., Warrens A.N. (2005). Attitudes toward transplantation in U.K. Muslim Indo-Asians in west London. Am. J. Transplant..

[10] Meyers D.G. (2005). Social Psychology.

[11] Batson C.D. (2011). Altruism in Humans.

[12] de Waal F.B. (2008). Putting the altruism back into altruism: The evolution of empathy. Annu. Rev. Psychol..

[13] Monday O.I. (2020). Is altruism always sufficient for organ donation? vroom’s expectancy theory, for expanding the organ donor pool. Saudi J. Kidney Dis. Transplant..

[14] Bekkers R. (2007). Measuring Altruistic Behavior in Surveys: The All-or-Nothing Dictator Game. Surv. Res. Methods.

[15] Carpenter J., Connolly C., Myers C.K. (2008). Altruistic behavior in a representative dictator experiment. Exp. Econ..

[16] Hilbig B.E., Thielmann I., Hepp J., Klein S.A., Zettler I. (2015). From personality to altruistic behavior (and back): Evidence from a double-blind dictator game. J. Res. Personal..

[17] Milaniak I., Wilczek-Rużyczka E., Przybyłowski P. (2018). Role of empathy and altruism in organ donation decision-making among nursing and paramedic students. Transplant. Proc..

[18] de Groot J.I.M., Steg L. (2008). Value orientations to explain beliefs related to environmental significant behavior: How to measure egoistic, altruistic, and biospheric value orientations. Environ. Behav..

[19] Rushton J.P., Chrisjohn R.D., Fekken G.C. (1981). The altruistic personality and the self-report altruism scale. Personal. Individ. Differ..

[20] Chou K.L. (1996). The Rushton, Chrisjohn and Fekken Self-Report Altruism Scale: A Chinese translation. Personal. Individ. Differ..

[21] Khanna R., Singh P., Rushton J.P. (1993). Development of the Hindi version of a Self-Report Altruism Scale. Personal. Individ. Differ..

[22] Aguilar Pardo D., Martínez Cotrina J. (2016). Validation of the self-report altruism scale test in Colombian University Students. Ánfora.

[23] Suseno M. (2019). Adapting self-report altruism scale to measure altruistic behavior of pre-service teachers in Indonesia. Int. J. Psychosoc. Rehabil..

[24] Karacan E., Cengiz Seval G., Aktan Z., Ayli M., Palabiyikoglu R. (2013). Blood donors and factors impacting the blood donation decision: Motives for donating blood in Turkish sample. Transfus. Apher. Sci..

[25] Garofalo C., Noteborn M., Sellbom M., Bogaerts S. (2019). Factor structure and construct validity of the Levenson self-report psychopathy scale (LSRP): A replication and extension in dutch nonclinical participants. J. Personal. Assess..

[26] Khalaila R. (2013). Religion, altruism, knowledge, and attitudes toward organ donation: A survey among a sample of Israeli college students. Med. Law.

[27] Newton J.D. (2011). How does the general public view posthumous organ donation? A meta-synthesis of the qualitative literature. BMC Public Health.

[28] Elalouf A., Pliskin J.S., Kogut T. (2020). Attitudes, knowledge, and preferences of the Israeli public regarding the allocation of donor organs for transplantation. Isr. J. Health Policy Res..

[29] Watad S.A. (2004). The Connection between Equality in Parental Relationships and Motives for Help and the Provision of Help among Adolescent Children. Doctoral Dissertation.

[30] Utitz L. (2002). Behavioral Beliefs, Attitudes toward Behavior, Normative Beliefs, Subjective Norms, Perception of Controlling Ability, and Behavioral Intent Regarding Organ Donation and Signing a Donor Card. Master’s Thesis.

[31] Perenc L., Radochonski M., Radochonski A. (2012). Knowledge and attitudes of Polish university students toward organ donation and transplantation. Psychol. Health Med..

[32] Radochonski M., Radochonski A. (2007). Attitudes of Polish university students toward voluntary blood donation. Prz. Med. Uniw. Rzesz..

[33] Robbins R.A. (1990). Signing an organ donor card: Psychological factors. Death Stud..

[34] Wu A.M., Tang C.S. (2009). The negative impact of death anxiety on self-efficacy and willingness to donate organs among Chinese adults. Death Stud..

[35] Cohen E.L., Hoffner C. (2013). Gifts of giving: The role of empathy and perceived benefits to others and self in young adults’ decisions to become organ donors. J. Health Psychol..

[36] Hill E.M. (2016). Posthumous organ donation attitudes, intentions to donate, and organ donor status: Examining the role of the big five personality dimensions and altruism. Personal. Individ. Differ..

[37] Kurleto P., Skorupska-Król A., Broniatowska E., Bramstedt K.A. (2020). Exploring the motives of Israeli Jews who were living kidney donors to strangers. Clin. Transplant..

[38] Mostafa M.M. (2010). Altruistic, cognitive, and attitudinal determinants of organ donation intention in Egypt: A social marketing perspective. Health Mark. Q..

[39] Roff S.R. (2007). Self-interest, self-abnegation, and self-esteem: Towards a new moral economy of non-directed kidney donation. J. Med. Ethics.

[40] Ghorbani F., Khoddami-Vishteh H.R., Ghobadi O., Shafaghi S., Louyeh A.R., Najafizadeh K. (2011). Causes of family refusal for organ donation. Transplant. Proc..

[41] López J.S., Soria-Oliver M., Aramayona B., García-Sánchez R., Martínez J.M., Martín M.J. (2018). An integrated psychosocial model of relatives’ decision about deceased organ donation (IMROD): Joining pieces of the puzzle. Front. Psychol..

[42] Akgün S., Tokalak I., Erdal R. (2002). Attitudes and behavior related to organ donation and transplantation: A survey of university students. Transplant. Proc..

[43] Saleem T., Ishaque S., Habib N., Hussain S.S., Jawed A., Khan A.A., Ahmad M.I., Iftikhar M.O., Mughal H.P., Jehan I. (2009). Knowledge, attitudes, and practices survey on organ donation among a selected adult population of Pakistan. BMC Med. Ethics.

[44] Uyar M., Demir L.S., Durduran Y., Evci R., Diker Ardıç Z., Şahin T.K. (2019). Patient Knowledge, Attitudes, and Behaviors Associated with Organ Donation. Ann. Transplant..

[45] Zambudío A.R., Martínez-Alarcón L., Parrilla P., Ramírez P. (2009). Attitude of nursing staff toward organ donation in a Spanish hospital with a solid-organ transplant program. Prog. Transplant..

[46] Fishbein M., Ajzen I. (1975). Belief, Attitude, Intention, and Behavior: An Introduction to Theory and Research.

[47] Krupic F., Westin O., Hagelberg M., Sköldenberg O., Samuelsson K. (2019). The Influence of Age, Gender and Religion on Willingness to be an Organ Donor: Experience of Religious Muslims Living in Sweden. J. Relig. Health.

[48] Febrero B., Ros I., Almela-Baeza J., Pérez-Sánchez M.B., Rodríguez J.M., Alconchel F., Ruiz-Manzanera J.J., Martínez-Insfran L.A., Domingo J., Martínez-Alarcón L. (2020). Attitude of Older People Toward Living Donation. Transplant. Proc..

[49] López J.S., Valentín M.O., Scandroglio B., Coll E., Martín M.J., Sagredo E., Martínez J.M., Serna E., Matesanz R. (2012). Factors related to attitudes toward organ donation after death in the immigrant population in Spain. Clin. Transplant..

[50] Carmack H.J., DeGroot J.M. (2020). Communication Apprehension About Death, Religious Group Affiliation, and Religiosity: Predictors of Organ and Body Donation Decisions. OMEGA-J. Death Dying.

[51] Bresnahan M.J., Guan X., Smith S.W., Wang X., Edmundson J.Z. (2010). Cultures of the soul: Spiritual beliefs about organ donation in China and the United States. Chin. J. Commun..

[52] Yoon S.M. (2019). Interaction Effects of Religiosity Level on the Relationship between Religion and Willingness to Donate Organs. Religions.

[53] Scott O., Jacobson E. (2007). Implementing presumed consent for organ donation in Israel: Public, religious and ethical issues. IMAJ.

[54] Dopelt K., Cohen D., Amar-Krispel E., Davidovitch N., Barach P. (2021). Facing Death: Attitudes toward Physician-Assisted End of Life among Physicians Working at a Tertiary-Care-Hospital in Israel. Int. J. Environ. Res. Public Health.

